# Synchronous Bladder Metastasis From Chromophobe Renal Cell Carcinoma: A Report of a Rare Case

**DOI:** 10.7759/cureus.86234

**Published:** 2025-06-17

**Authors:** Jose L Romero Uribe, Victor Cornejo Davila, Jose Cruz Ruiz

**Affiliations:** 1 Urology, Hospital General de Culiacán, Culiacán, MEX; 2 Urology, Hospital Regional de Alta Especialidad "Ciudad Salud", Tapachula, MEX

**Keywords:** bladder metastasis, chromophobe, nephrectomy, partial cystectomy, renal cell carcinoma

## Abstract

Renal cell carcinoma (RCC) is the most frequent form of kidney cancer and represents a small percentage of all adult cancers. Metastasis of RCC to the bladder is exceptionally uncommon, with only a limited number of cases documented, the majority being of the clear cell subtype. Instances involving the chromophobe variant are particularly rare.

We report the case of a 29-year-old female presenting with anemia, gross hematuria, and left flank pain. Abdominal computed tomography (CT) revealed a left renal mass with features suggestive of a perinephric abscess, along with a bladder lesion. Cystoscopy with transurethral resection of the bladder tumor (TURBT) and percutaneous drainage of the abscess were performed, followed by left radical nephrectomy. Histopathological examination of both renal and bladder specimens confirmed the eosinophilic variant of chromophobe RCC (chRCC). A partial cystectomy was subsequently performed, achieving R0 resection.

This case highlights an unusual metastatic pattern of chRCC and underscores the importance of aggressive surgical management in selected patients.

## Introduction

Renal cell carcinoma (RCC) is a heterogeneous group of malignancies arising from the renal epithelium and accounts for approximately 2-3% of all adult malignancies. Among its histologic subtypes, the chromophobe variant (chRCC) represents 5-7% of RCC cases and typically demonstrates a more indolent course than the most common clear cell RCC [1,2]. Despite its generally favorable prognosis, chRCC can occasionally metastasize. The eosinophilic variant of chRCC is characterized by distinct histological features, including abundant granular eosinophilic cytoplasm, prominent cell borders, and perinuclear clearing or halos. While it shares these morphological traits with oncocytoma, it can be distinguished by cytogenetic abnormalities and immunohistochemical markers such as CK7 and CD117 positivity. Despite its unique histologic appearance, it generally shares a similar prognosis with the classic variant [3].

Metastases are present in approximately one-third of patients with RCC at the time of diagnosis, and an additional one-third develop metastatic disease during follow-up [2]. The bladder is an exceptionally rare site of dissemination, comprising less than 2% of all RCC metastases [4,5]. Bladder metastases are more commonly associated with the clear cell subtype. Synchronous bladder metastases of chRCC are exceedingly rare, with only isolated cases described in the literature [6,7]. We report a rare case of synchronous bladder metastasis from chRCC in a young woman.

## Case presentation

A 29-year-old female presented with a two-year history of chronic left flank pain. She also reported episodes of gross hematuria with clot formation, resulting in anemia. Constitutional symptoms included fatigue and intermittent fever. Physical examination revealed pallor and tenderness over the left costovertebral angle. Laboratory findings demonstrated severe anemia (hemoglobin: 5.1 g/dL), leukocytosis, gross hematuria, and evidence of acute kidney injury (AKI stage I) with a serum creatinine of 1.9 mg/dL (Table 1).

**Table 1:** Initial laboratory findings.

Test	Parameter	Measured value	Units	Reference range
Blood work	Hemoglobin	5.1	mg/dL	13.0-18.0
	RBC	15	x10^6^/uL	4.70-6.10
	WBC	18.6	x10^3^/uL	4.0-11.03
	Platelets	395	x10^3^/uL	140.0-450.0
	Glucose	92	mg/dL	70-100 (fasting)
	Creatinine	1.9	mg/dL	0.60-1.30
	Blood urea nitrogen	40.0	mg/dL	8.4-25.7
	Prothrombin time (PT)	15	sec	9.4-12.5
	Activated partial thromboplastin time (APTT)	28	sec	28.1-39.7
	International normalised ratio (INR)	1.3	INR	0.8-1.2
Urinalysis	Appearance	Red	-	Clear, pale, yellow, amber
	Protein	Negative	-	Negative
	Blood	>100 RBCs	-	0-3
	Leukocytes	20-30 WBCs	-	0-2
	Nitrite	Negative	-	Negative

**Figure 1:**
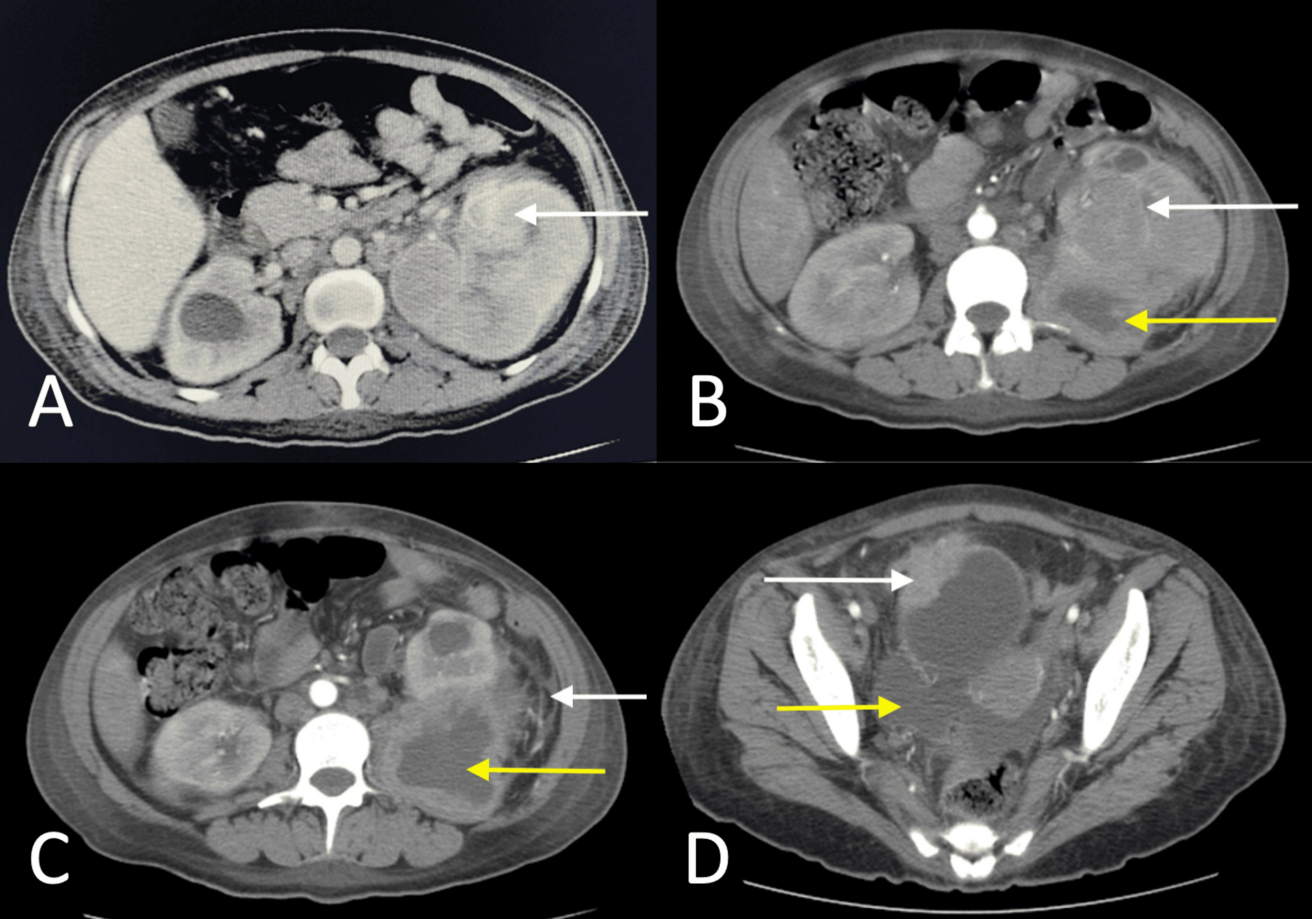
Contrast-enhanced axial CT images in the corticomedullary (A) and nephrographic phases (B, C, D). (A, B) Heterogeneous left renal mass primarily involving the mid and lower poles, with extension toward the upper pole (white arrows). The lesion shows heterogeneous enhancement, increasing from 15 to 50 Hounsfield Units (HU), with central non-enhancing areas suggestive of necrosis. The mass measures approximately 8.5 × 7 × 5 cm. Retroperitoneal lymphadenopathy is present. No definite evidence of renal vein invasion is seen. Abscess formation is noted in the lower pole (yellow arrow). (C) Perinephric fat stranding (white arrow) and abscess formation in the lower pole of the left kidney (yellow arrow). (D) Bladder tumor involving the right lateral wall with perivesical extension (white arrow), accompanied by ascites (yellow arrow).

A contrast-enhanced CT urography revealed an atypical heterogeneous left renal mass with perinephric fat stranding and abscess, and an intravesical tumor on the right bladder wall (Figure 1). No metastases to the lungs or liver were detected on staging CT.

**Figure 2:**
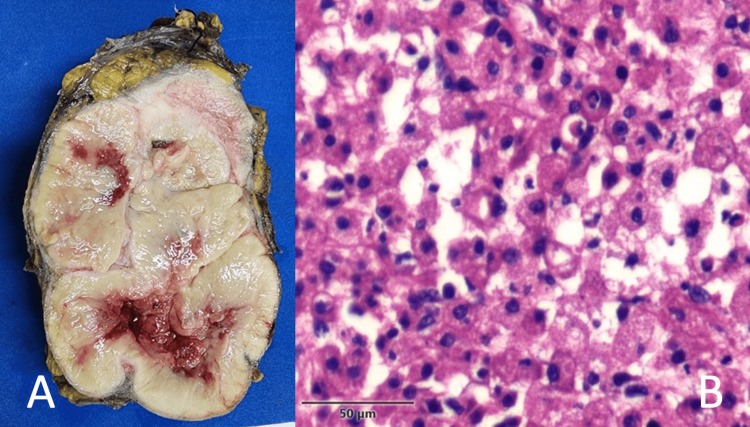
(A) Gross specimen of the left kidney showing a well-circumscribed tan-pink mass with central hemorrhage and necrosis. (B) Histological section (H&E stain, 400×) showing polygonal tumor cells with prominent cell borders, perinuclear clearing, and pale eosinophilic cytoplasm, consistent with the eosinophilic variant of chromophobe renal cell carcinoma (RCC).

Initial management included transfusion of two units of packed red blood cells, resulting in a hemoglobin level of 9.5 g/dL, along with administration of broad-spectrum antibiotics. The patient underwent cystoscopy with transurethral resection of the bladder tumor (TURBT) and percutaneous drainage of the perinephric abscess. By postoperative day 6, she was asymptomatic with clinical improvement. Abscess culture grew *Escherichia coli* sensitive to ertapenem, and a 10-day course of antibiotics was completed. A left radical nephrectomy was subsequently performed. Histopathological analysis revealed an eosinophilic variant of chRCC extending into the perinephric tissues, consistent with pT3a disease. Three regional lymph nodes, two paraaortic and one hilar, were positive for metastatic involvement (pN1), and similar histopathological features were observed in the bladder biopsy (Figure 2).

**Figure 3:**
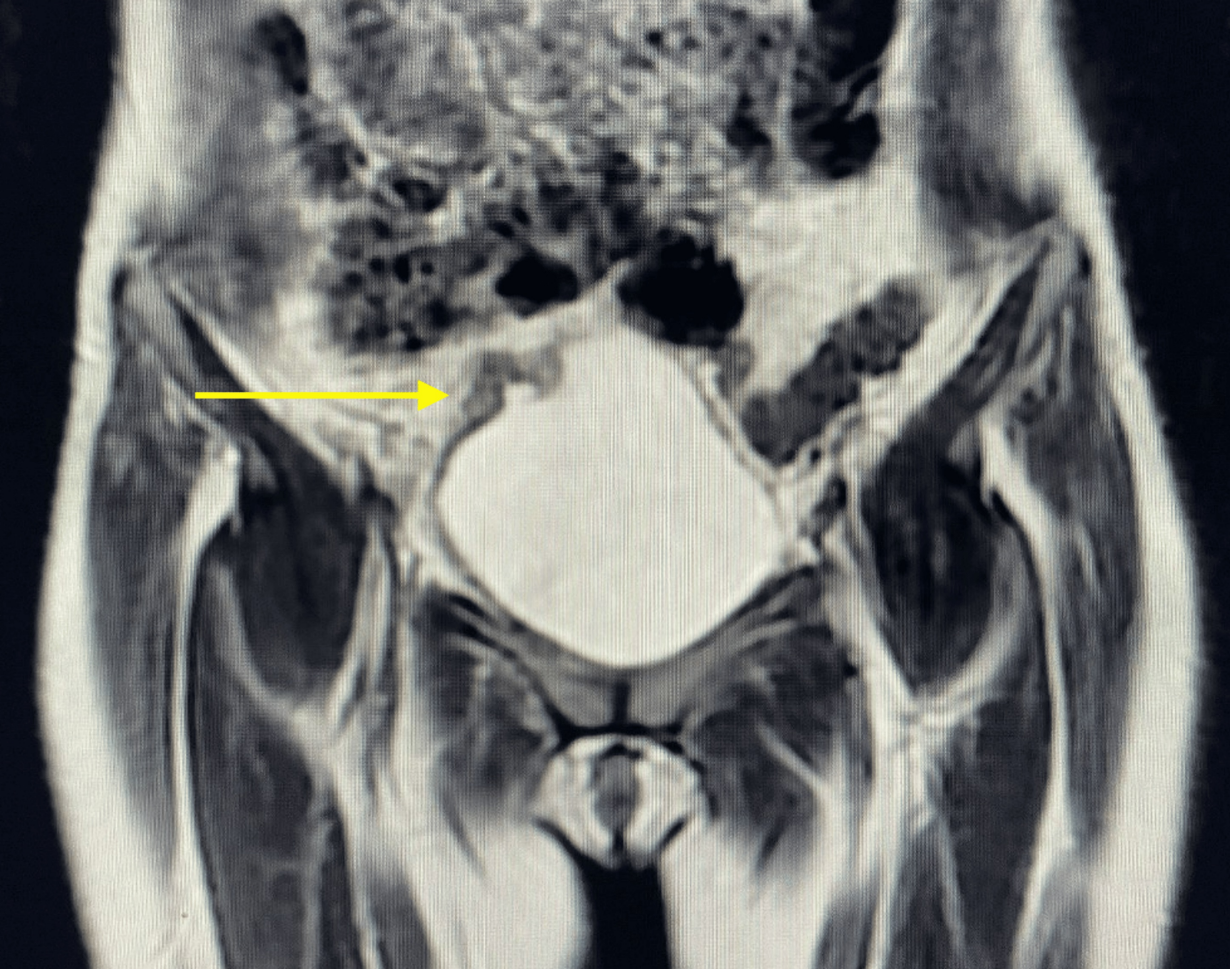
Coronal T2-weighted MRI demonstrating a large intravesical mass arising from the right lateral bladder wall. The lesion appears hyperintense relative to muscle, with well-defined borders and mass effect on the bladder lumen, consistent with bladder metastasis (yellow arrow).

Given the absence of systemic disease and a favorable postoperative course, an MRI was performed prior to the planned partial cystectomy (Figure 3), and the intervention was deferred for two months. According to the treating urologist, the decision to delay surgical management of the bladder lesion was based on concerns that performing both the radical nephrectomy and the transurethral resection of the bladder lesion in a single operative session could significantly increase surgical morbidity. Therefore, the procedures were staged to minimize perioperative risk and ensure optimal patient safety. Nonetheless, concurrent surgeries may be feasible in appropriately selected patients.

**Figure 4:**
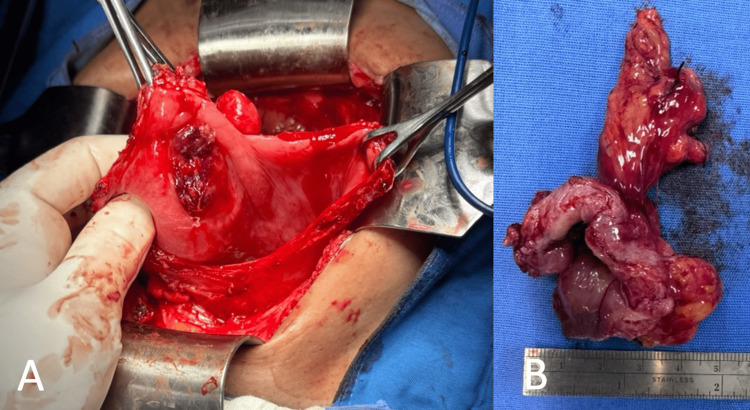
(A) Intraoperative view during partial cystectomy showing an infiltrating sessile bladder tumor. (B) Gross specimen from the partial cystectomy.

**Figure 5:**
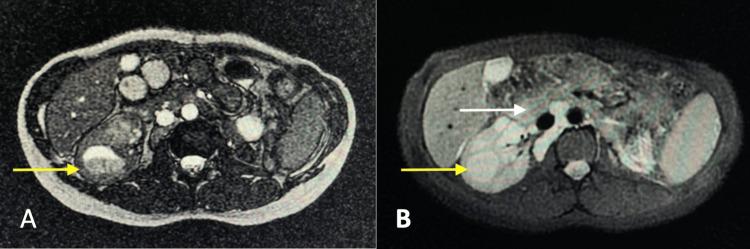
(A) Balanced turbo field echo (BTFE) MRI sequence showing a left renal mass with heterogeneous signal intensity (yellow arrow). (B) Diffusion-weighted MRI (b-0) demonstrating the same renal mass (yellow arrow) along with enlarged retroperitoneal lymph nodes (white arrow), suggestive of disease progression from renal cell carcinoma.

Histological examination confirmed metastatic chRCC with extension into perivesical fat but negative margins (R0 resection) (Figure 4). Final staging was pT3a pN1 M1. The patient remained asymptomatic but was lost to follow-up due to her marginalized situation, including poor economic status and a long distance from the medical center. She eventually developed abdominal pain, systemic symptoms, and renal failure. MRI revealed disease progression, demonstrating retroperitoneal lymph node conglomerates and a right renal mass, which may suggest progression of RCC metastasis (Figure 5). The patient was managed expectantly without adjuvant therapy, and no evaluation for genetic cancer syndromes was performed. The patient died nine months after the initial diagnosis.

## Discussion

Bladder metastasis from RCC is rare, more often seen in the metachronous setting, with a median interval of 33 months after nephrectomy [8,9]. The pathophysiology of bladder involvement remains unclear. Proposed mechanisms include hematogenous dissemination, retrograde lymphatic or venous spread, and direct intraluminal tumor cell seeding. In this case, hematogenous dissemination appears to be the most plausible route, given the absence of contiguous spread, the vascular nature of RCC, and the isolated bladder lesion without evidence of regional lymphatic involvement [10,11].

Most reported cases involve the clear cell subtype. Metastases from chRCC are even less common and may present with hematuria or urinary obstruction, depending on the site involved in the bladder [12].

There is no standardized treatment protocol for bladder metastases of RCC due to their low frequency; however, the same principles for metastatic RCC treatment are applied, with aggressive surgical resection, if feasible, and systemic targeted therapies, if available. In the bladder, treatment options usually involve partial or radical cystectomy because metastases usually involve the muscle and not just the epithelium [5,6], such as this case. Complete surgical excision can improve short-term outcomes in patients with isolated metastases [4,6].

Targeted therapies, including vascular endothelial growth factor (VEGF) and platelet-derived growth factor (PDGF) inhibitors, as well as immune checkpoint inhibitors (ICIs), have demonstrated effectiveness in metastatic RCC, particularly in tumors with clear cell histology [2]. However, evidence supporting their efficacy in chRCC is limited due to its rarity and biological differences [13]. However, the benefit of systemic therapy in chRCC remains less clear. In selected patients with isolated metastasis and good performance status, observation after complete surgical resection might be an option [6,8], but we should keep in mind that complete resection can result in effective disease control. In this case, even though R0 resection was achieved, the disease progressed in less than a year, which probably highlighted the aggressiveness of this presentation at such a young age.

Although evidence is limited, everolimus-based treatments appear to show the most promising activity in patients with chRCC. In the ASPEN phase II trial, everolimus monotherapy in metastatic chRCC (non-clear cell) achieved a 33% objective response rate (ORR) (two out of six patients responded) [14]. Responses to ICI monotherapy or ICI-based combination therapies are variable; it has shown limited efficacy in chRCC, suggesting potential resistance to ICI, but selected patients may benefit. No statistically significant difference has been found regarding overall survival when comparing sunitinib and everolimus [13]. Evidence for chRCC is limited, but it may offer opportunities for future targeted therapies.

This case is remarkable due to the synchronous metastasis presentation of a less common variant, the bladder involvement with no other obvious sites of metastases, and the patient's young age. This case also highlights the importance of fully assessing symptoms such as flank pain in young patients, especially if it’s accompanied by systemic symptoms or hematuria; even though most etiologies will be benign in this age group, other serious underlying conditions, including malignancies, can be diagnosed as well. The case shows the need to provide better healthcare to marginalized patients as well, since it can affect the disease outcome.

## Conclusions

Bladder metastasis from chRCC is extremely rare and presents significant diagnostic and therapeutic challenges. Complete surgical resection of both primary and metastatic lesions has been associated with improved outcomes, particularly in cases of isolated metastasis. This case highlights the need for awareness of atypical metastatic patterns and supports aggressive surgical intervention as a viable treatment strategy in carefully selected patients. It also contributes to the limited body of literature on this topic and underscores the importance of a thorough evaluation in patients presenting with hematuria and renal masses.
